# Identification of hypertensive patients with dominant affective temperaments might improve the psychopathological and cardiovascular risk stratification: a pilot, case–control study

**DOI:** 10.1186/s12991-015-0072-0

**Published:** 2015-10-26

**Authors:** Andrea László, Levente Babos, Zsóka Kis-Igari, Adrienn Pálfy, Péter Torzsa, Ajándék Eőry, László Kalabay, Xenia Gonda, Zoltán Rihmer, Orsolya Cseprekál, András Tislér, Judit Hodrea, Lilla Lénárt, Andrea Fekete, János Nemcsik

**Affiliations:** Department of Family Medicine, Semmelweis University Budapest, Budapest, Hungary; Department of Pharmacodynamics, Semmelweis University Budapest, Budapest, Hungary; Department of Clinical and Theoretical Mental Health, Semmelweis University Budapest, Budapest, Hungary; MTA-SE Neurochemistry Research Group, Budapest, Hungary; Ist Department of Internal Medicine, Budapest, Hungary; MTA-SE “Lendület” Diabetes Research Group Budapest, Budapest, Hungary; Health Service of Zugló (ZESZ), Budapest, Hungary

**Keywords:** Hypertension, Affective temperaments, Depression, Anxiety, Arterial stiffness, Brain-derived neurotrophic factor, Cardiovascular risk

## Abstract

**Background:**

Although mood disorders and cardiovascular diseases have widely studied psychosomatic connections, data concerning the influence of the psychopathologically important affective temperaments in hypertension are scarce. To define a possibly higher cardiovascular risk subpopulation we investigated in well-treated hypertensive patients with dominant affective temperaments (DOM) and in well-treated hypertensive patients without dominant temperaments the level of depression and anxiety, arterial stiffness and serum Brain-derived Neurotrophic Factor (seBDNF).

**Methods:**

175 hypertensive patients, free of the history of psychiatric diseases, completed the TEMPS-A, Beck Depression Inventory and Hamilton Anxiety Scale questionnaires in two primary care practices. Of those 175 patients, 24 DOM patients and 24 hypertensive controls (matched in age, sex and the presence of diabetes) were selected for measurements of arterial stiffness and seBDNF level.

**Results:**

Beck and Hamilton scores in DOM patients were higher compared with controls. Pulse wave velocity and augmentation index did not differ between the groups while in the DOM patients decreased brachial systolic and diastolic and central diastolic blood pressures were found compared with controls. SeBDNF was lower in the DOM group than in the controls (22.4 ± 7.2 vs. 27.3 ± 7.8 ng/mL,* p* < 0.05).

**Conclusions:**

Although similar arterial stiffness parameters were found in DOM patients, their increased depression and anxiety scores, the decreased brachial and central diastolic blood pressures as well as the decreased seBDNF might refer to their higher vulnerability regarding the development not only of major mood disorders, but also of cardiovascular complications. These data suggest that the evaluation of affective temperaments should get more attention both with regard to psychopathology and cardiovascular health management.

## Background

Mood disorders are common public health problem in the Western world and their strong connection to cardiovascular diseases (CVD) is well known [1]. Moreover, the negative impact of adverse individual psychological traits and characteristics, like anger, hostility and anxiety is also well-documented in connection to the development and progression of coronary heart disease [2, 3], while individual differences in antagonism-related traits seem to predict a variety of cardiovascular outcomes [4, 5].

There are multiple mechanisms by which depression could increase the probability of vascular diseases, such as increased platelet activation [6], inflammatory markers [7] as well as reduced heart rate variability [8]. Parallel to this, well-established biomarkers of inflammation were also found to be elevated in persons in states of anger or hostility [9, 10], while a reduced function of the autonomic nervous system was observed in states of anxiety [11] and interpersonal antagonism was connected with carotid arterial intima-media thickening [12].

Another possible link might be the brain-derived neurotrophic factor (BDNF), a member of the neurotrophic factor family, which is not only associated with major depressive disorder [13], but its higher serum level is also connected to a decreased risk of cardiovascular disease and mortality [14].

The independent continuous relationship between elevated blood pressure and several cardiovascular events is well-known for patients in all ages and ethnic groups [15]. Besides elevated blood pressure, arterial stiffening is increasingly recognised as a risk factor for CVD. The determination of decreased arterial elasticity helps to identify the patient’s higher risk of cardiovascular morbidity and mortality [16]. In Europe, the measurement of arterial stiffness is already a recommended method for cardiovascular risk assessment among hypertensive patients [17]. Previously, depressive symptoms, especially in diagnosed depressive disorders were found to be associated with increased arterial stiffening [18].

According to our recent findings, specific affective temperament types (depressive, cyclothymic, hyperthymic, irritable and anxious) are the subclinical, trait-related manifestations and commonly the antecedents of minor and major mood disorders [19]. Recently, in a study of primary care patients we also demonstrated a strong connection between dominant cyclothymic temperament and hypertension [20]. Moreover, we also showed that cyclothymic temperament is connected to acute coronary events in hypertensive patients [21].

We hypothesised that hypertensive patients with dominant affective temperaments (DOM) score higher depression and anxiety values and have impaired arterial stiffness, central blood pressure or serum BDNF compared with hypertensive patients without dominant affective temperaments, forming a high-risk subgroup patient population.

## Methods

### Patients

In our cross-sectional case–control study well-treated chronic (>12 months medication) hypertensive Caucasian patients were investigated in two primary care practices. All patients completed the TEMPS-A, Beck Depression Inventory (BDI) and Hamilton Anxiety Scale (HAM-A) questionnaires in order to evaluate the presence of affective temperaments, depression and anxiety, respectively. Following this initial screening, patients with dominant affective temperaments (reaching the mean + 2 SD point scores or higher in each affective temperament subscale, DOM) were identified. Hypertensive controls without DOM, matched in age, gender and presence of diabetes, were selected from the initial hypertensive patient cohort and included in the arterial stiffness and seBDNF measurements. As blood pressure medication can highly influence arterial stiffness, it was further analysed, but patients were not matched in this aspect.

Exclusion criteria were the history or ongoing treatment of depression or anxiety (as with arterial stiffening the associations are clarified [18]), and the presence of atrial fibrillation or uncontrolled hypertension (>145/95 mmHg in repeated office measurements). In patients with an average blood pressure between 140/90 and 145/95 mmHg in repeated office blood pressure measurements, ambulatory 24-h blood pressure monitoring or home blood pressure monitoring was performed and only well-controlled patients were admitted to the study. Prior to the participation, all patients gave their written informed consent. The study was approved by the Scientific and Research Ethics Committee of the Medical Research Council, Hungarian Ministry of Health (ETT TUKEB 842/PI/2011) and carried out in accordance with the tenets of the Declaration of Helsinki.

### Procedures

During the initial visit patients completed the questionnaires. Physical examination (blood pressure, heart rate, height, weight and waist circumference) were completed and data on medical history (with special attention to cardiovascular risk factors, complications and depression) as well as on current medication was collected.

After the analysis of the questionnaires, patients meeting the criteria of dominant temperament and their controls were invited for arterial stiffness and seBDNF measurements, which took place within 1 month after the initial visit.

### Questionnaires

*The temperament evaluation of Memphis Pisa, Paris and San Diego (*TEMPS-A) questionnaire is an 110-item, self-report instrument, developed to measure affective temperaments on depressive, cyclothymic, hyperthymic, irritable and anxious subscales and requiring the answers ‘yes’ (score 1) or ‘no’ (score 0) [22]. TEMPS-A is used to assess the point scores of each subscale and also to measure the presence of the dominant form of affective temperaments by taking the mean of the subscale and adding up two standard deviations to it. Those reaching the mean + 2 SD level or higher in each subscale are considered to have dominant affective temperaments.

The *beck depression inventory* (BDI), created by Aaron T. Beck, is a 21-question multiple-choice self-report questionnaire, one of the widely used instruments for measuring the severity of depression. Participants are asked to make ratings on a four-point scale, where a higher score correlates with more severe depression.

*Hamilton Anxiety Scale* (HAM-A) was evaluated by the examiner to study the severity of anxiety. The scale consists of 14 items, each item is scored on a scale of 0 (not present) to 4 (severe anxiety).

### Arterial stiffness recordings

Measurements were performed in a temperature-controlled room in supine position, on the day of blood sampling, before it, between 7.00 and 8.00 a.m. Patients were required to fast overnight and refrain from smoking and caffeine-containing beverages before the procedure, but to take the usual blood pressure regulating medication. Upon arrival at the investigation unit, the subjects were equipped with measurement devices, and then rested in supine position for approximately 15 min before being measured. Arterial stiffness parameters were evaluated using the validated, gold-standard PulsePen tonometer (DiaTecne, Milan, Italy, [23]). With this method the pulse wave velocity (PWV) augmentation index (AI), as well as the central systolic blood pressure (central SBP) and central pulse pressure (central PP) can be calculated. In each subject two sequences of measurements were performed and their mean was used for statistical analysis. ECG was recorded continuously from the limb lead with the largest R wave.

In the PWV calculations 80 % of the carotid-femoral distance was used, following the recent guideline [24]. Previously we evaluated the intra- and interobserver variabilities of PWV measurements obtained by the PulsePen device on hypertensive patients and found them to be 4.6 and 6.3 % high, respectively. As PulsePen calculates pressure values using brachial diastolic blood pressure calibration, the calculated central and brachial diastolic blood pressure values were identical [23].

### Measurement of seBDNF concentration

Peripheral blood samples of patients were collected in anticoagulant-free tubes, right after the measurement of arterial stiffness. After centrifugation (3600 rpm for 6 min) the serum was stored at −20 °C. SeBDNF was measured using commercially available sandwich enzyme-linked immunosorbent assay (R&D Systems, Minneapolis MN, USA) according to the manufacturer’s protocol and serum BDNF level was determined in ng/mL.

### Data analysis

Data were expressed as mean ± SEM and medians. Differences in variables between controls and DOM patients were analysed using unpaired Student’s *t* tests or Mann–Whitney rank-sum tests for data failing tests of normality. Blood pressure medications were calculated and compared using equivalent doses, differences were analysed with unpaired Student’s *t* tests or Mann–Whitney rank-sum tests. Significance was accepted at *p* < 0.05. Statistical analysis was performed using the SigmaStat for Windows Version 3.5 (SPSS) program package.

## Results and discussion

### Characteristics of the patients

183 hypertensive patients were recruited to participate in the study and 175 of them completed the questionnaires. 29 of the patients had dominant affective temperaments (DOM). Out of the 29 DOM patients one died 3 days prior to the planned arterial stiffness measurement and four declined to further participate in arterial stiffness measurements. The arterial stiffness and BDNF of altogether 48 hypertensive patients was evaluated: 24 DOM and 24 control subjects matched in age, gender and presence of diabetes.

Among the DOM patients six subjects with depressive, five irritable and four anxious dominant temperaments were found. In the other patients, combinations of dominant temperaments were present: three patients had cyclothymic and depressive, two had cyclothymic and irritable, two had cyclothymic, depressive and anxious, one had cyclothymic, irritable and anxious temperaments and one patient was dominant for cyclothymic, irritable, anxious and depressive affective temperaments. No patient with a dominant hyperthymic temperament was found in our cohort.

### Comparing the control and DOM patients for statistical differences

Baseline demographic, anthropometric and laboratory parameters and the used cardiovascular medications of the patients are presented in Table 1. SeBDNF levels were lower in DOM patients. Compared with controls, beta-blockers were prescribed more frequently and in higher dose in the DOM group [0.63 (0–5) vs. 5 (0.63–6.88) mg, calculated for bisoprolol, respectively, *p* < 0.05]. No differences were found in the mean duration of hypertension among controls and DOM patients [10.5 (4–15.8) vs. 12 (6.5–17.8) *p* = 0.238, respectively]. No differences were found among the groups studied in smoking habits and in the frequency of physical training (data are not shown).

**Table 1:** Baseline demographic, anthropometric and laboratory parameters and the used cardiovascular medication of the patients

	Control	DOM
*N* (male:female)	24 (9:15)	24 (9:15)
Age (year)	63.7 ± 2.54	64.3 ± 2.52
Body height (cm)	164.5 ± 1.51	166 ± 2.06
Body weight (kg)	76.3 ± 2.66	81 ± 3.34
AC (cm)	101.5 ± 4.1	102 ± 5.5
BMI (kg/m^2^)	28.2 (26–31.2)	29.8 (24.4–34.6)
Glucose (mmol/l)	5.36 (5.03–6.07)	5.7 (4.94–6.63)
CKD-EPI GFR (mmol/l)	77.5 (62.5–86)	78.5 (60.8–90)
Uric acid (µmol/l)	309.3 ± 15.26	321.6 ± 16.82
Cholesterol (mmol/l)	5.48 ± 0.22	5 ± 0.35
Triglyceride (mmol/l)	1.36 (1.08–2.18)	1.82 (0.99–2.15)
BDNF (ng/ml)	27.29 (24.2–30.35)	20.10 (14.74–27.11)*
ACE-inhibitors [n (%)]	13 (54.2)	13 (54.2)
ARBs	9 (37.5)	5 (20.8)
Calcium-channel blockers	11 (45.8)	11 (45.8)
Beta-blockers	12 (50)	18 (75)*
Diuretics	11 (45.8)	11 (45.8)
Antiplatelet medication	8 (33.3)	11 (45.8)

The values are mean ± SEM or medians (quartiles)

The groups were compared for differences by using Student’s *t* test or the Mann–Whitney rank-sum for data failing tests of normality

*DOM* patients with dominant affective temperament, *AC* abdominal circumference, *BMI* body mass index, *CKD-EPI GFR* glomerular filtration rate assessed by the chronic kidney disease epidemiology collaboration glomerular filtration rate equation; BDNF: brain-derived neurotrophic factor, *ARBs* angiotensin II receptor blockers

* *p* < 0.05 compared with controls

Table 2 represents the differences in the five affective temperaments and in the BDI and HAM-A scores. Compared with controls, in DOM patients depressive, cyclothymic, irritable and anxious scores were higher, while hyperthymic scores were equal. Both BDI and HAM-A scores were markedly higher in DOM patients than in the controls.

**Table 2:** TEMPS-A scores of affective temperaments, BDI and HAM-A scores

	Control *N* = 24	DOM *N* = 24
Depressive	6 (4.25–8.75)	12.5 (7–13.75)*
Cyclothymic	3 (1–5)	9.5 (7–13.75)*
Hyperthymic	12.13 ± 0.73	10.46 ± 0.99
Irritable	2 (1–4.75)	9.5 (5–11)*
Anxious	5.08 ± 0.81	14.67 ± 0.88*
BDI	5 (2–7.75)	14.5 (8.5–19.75)*
HAM-A	7 ± 1.27	16.67 ± 1.68*

The values are mean ± SEM and medians (quartiles). The groups were compared for differences by using Student’s *t* test or the Mann–Whitney rank-sum for data failing tests of normality

*DOM* patients with dominant affective temperament, *TEMPS-A* the temperament evaluation of Memphis Pisa, Paris and San Diego questionnaire, *BDI* beck depression inventory, *HAM-A* Hamilton Anxiety Scale

* *p* < 0.05 compared with controls

Brachial and central hemodynamic and arterial stiffness parameters are shown in Table 3. Brachial systolic and both brachial and central diastolic and mean blood pressures were lower in DOM patients. PWV and AI, the two main arterial stiffness parameters, did not differ when compared with controls.

**Table 3:** Brachial and central hemodynamic and arterial stiffness parameters

	Control *N* = 24	DOM *N* = 24
HR (1/min)	66 ± 1.25	63.1 ± 1.17
SBPB (Hgmm)	130.8 (122–137.1)	122.6 (116.4–129.7)*
DBPB (Hgmm)	71.2 ± 1.5	66.6 ± 1.71*
MBPB (Hgmm)	91.9 ± 1.53	86.6 ± 1.94*
PPB (Hgmm)	59.3 (49.4–67.5)	54.3 (50.1–61.9)
Central SBP (Hgmm)	124.3 (113.5–136.4)	117 (112.6–131.7)
Central DBP (Hgmm)	71.2 ± 1.5	66.5 ± 1.71*
Central MBP (Hgmm)	94.1 ± 1.55	88.9 ± 1.96*
Central PP (Hgmm)	50.6 (44.5–66.6)	52.3 (48.8–1.72)
PWV (m/s)	9.32 (8.02–11.25)	8.74 (8.32–9.87)
AI (%)	16.04 ± 2.24	14.54 ± 2.66

The values are mean ± SEM and medians (quartiles). The groups were compared for differences by using Student’s t test or the Mann–Whitney rank-sum for data failing tests of normality

*DOM* patients with dominant affective temperament, *HR* heart rate, *SBPB* systolic brachial blood pressure, *DBPB* diastolic brachial blood pressure, *MBPB* mean brachial blood pressure, *PPB* brachial pulse pressure, *Central SBP* central systolic blood pressure, *Central DBP* central diastolic blood pressure, *Central MBP* central mean blood pressure, *Central PP* central pulse pressure, *PWV* pulse wave velocity, *AI* Augmentation index

* *p* < 0.05 compared with controls

Our pilot study was performed on hypertensive patients with dominant cyclothymic, irritable, depressive or anxious affective temperaments without the history or any present psychiatric medications. We found that DOM patients had higher anxiety and depression scores and lower seBDNF level. DOM patients had similar levels of arterial stiffening with a lower peripheral and central blood pressure compared with hypertensive controls.

### Affective temperaments are associated with depression and anxiety

Affective temperaments are tightly related to affective disorders. Hyperthymic temperament was found to be associated with type-I and cyclothymic with type-II bipolar disease [25] and atypical depression [19, 26], depressive temperament, in contrast to this, with unipolar major depression [19, 27]. In our cohort DOM patients also had higher depression and anxiety scores suggesting the importance of a joint evaluation of affective temperaments together with depression and anxiety even in those hypertensive patients who have no records of previous psychiatric diseases or any present antidepressant or anxiolytic medication.

### Affective temperaments and cardiovascular risk

Although the presence of dominant affective temperaments frequently precedes the onset of minor and major affective illness [19], and therefore screening for their presence would be an important target in the prevention and early intervention of cardiovascular disorders as well, only few studies are available which aim to investigate the role of affective temperaments in the development and risk of different cardiovascular diseases. Patients with depressive temperament were found to have worse metabolic control in type-2 diabetes [28], while cyclothymic, irritable and anxious temperaments showed affinity to obesity [29]. Recently we evaluated the role of affective temperaments in primary hypertension and found a significant association with the dominant cyclothymic temperament [20]. Moreover, cyclothymic temperament was associated with acute coronary events in hypertensive patient population [21]. In our present study, patients with affective temperaments had lower brachial and central diastolic and mean blood pressure values than controls. It is well known that decreased diastolic blood pressure is associated with increased mortality [30]. Parallel to our previous findings in hypertensive patients mentioned above [20, 21], this phenomenon of decreased blood pressure values might be more expressed in cyclothymic patients and would in this case suggest their increased susceptibility to cardiovascular complications. However, the clarification of this hypothesis requires further studies.

As our DOM patients had higher depression score compared with control patients, one explanation of the decreased blood pressure might reflect the fact that lower blood pressure levels are often accompanied by a pronounced presence of depressive symptoms and in follow-up studies symptoms of anxiety and depression predicted the development of lower blood pressure [31, 32]. In case of a co-occurring onset of depression and hypotension, the pathophysiological mechanisms are considered to be the alterations in neurohormonal, immune and autonomic regulations [33]. Whether only the increased depression per se caused the decreased blood pressure in our DOM patients, or another independent factor is also involved, is a question still to be answered.

Most of the studies support the idea that antagonistic traits, depression and anxiety increase the probability of the development of cardiovascular diseases [12, 34, 35], and data are also available with respect to arterial stiffness. In their population-based, cross-sectional study including 3704 elderly patients, Tiermeier et al. found that patients with increased arterial stiffness were more likely to have depressive symptoms. The association was stronger in cases with diagnosed depressive disorders. The authors concluded that arterial stiffness may partly cause the proposed relationship between vascular factors and depression [18]. In contrast to this we found no difference in PWV or augmentation index between our DOM and control hypertensive patients. An explanation to this phenomenon can be that in the study of Tiermeier et al. the patients’ arterial stiffness was much higher, as the border of the lowest PWV quartile was 11.4 m/s. This suggests a very poor vascular status of those patients, while in our patients PWV values were much lower.

In a recent study of Seldenrijk et al., depression and anxiety sensitivity and their association with arterial stiffening were evaluated. The authors found that out of these only anxiety sensitivity was associated with arterial stiffness; however, they studied only the augmentation index, which is a more variable parameter compared to PWV and is influenced by resistance vessels, that can be dysfunctional in patients with anxiety [36, 37]. It is also worth mentioning that the population of Seldenrijk et al. was much younger (46 years) compared with ours and only 18 % of the patients regularly took antihypertensive medication which suggests their better general resistance vessel function. Considering all these results we suppose that in older patients besides optimal vascular therapy, the deleterious effects of depression and anxiety for arterial stiffening can be attenuated, but in younger population without vascular medication the deleterious effects of anxiety can lead to detectable dysfunction of resistance vessels in comparison to healthy controls.

### Serum BDNF level, affective temperaments and allostatic load

In our study seBDNF, too, was measured and we observed its decreased level in our DOM patients. SeBDNF was found to be lower in patients suffering from major depressive disorder and some types of anxiety disorders, while in animal models the regulation of BDNF was suggested to contribute both to anxiety-like behaviour and hypertension [38–40]. BDNF was found to be decreased also in such pathological conditions as acute coronary syndrome and type 2 diabetes [41, 42]. Whether the decreased seBDNF in our hypertensive DOM patients is correlated with their higher depression and anxiety and bears any clinical relevance with respect to the cardiovascular outcome or not, further studies need to clarify.

The term “allostatic load” refers to a cumulative, multisystem view of the physiologic toll that is required for adaptation to stress. In mood disorders, especially in bipolar disorder allostatic load increases progressively as mood episodes occur over time [43]. Among many mediators, neurotrophic factors such as decreased level of BDNF indicates allostatic load [44]. Seeman et al. found in their longitudinal, community-based study that allostatic load may play a role in cardiovascular disorders [45] and there is evidence that reduction in allostatic load is associated with lower all-cause mortality, even in geriatric patients [46]. As affective temperaments are the subclinical manifestations of minor and major mood disorders and we found decreased BDNF level in patients with dominant temperaments, it can indicate an increased allostatic load among them, as well. According to this theory the increased cardiovascular risk could also be explained with the phenomenon of allostatic load.

In our study the depressive, cyclothymic, irritable and anxious temperaments or their combinations were investigated together. The neurobiological background of these temperaments seems to be at least partly common, as all were found to be associated with the 5-HTTLPR polymorphism of the serotonin transporter gene, namely the presence of the s allele, which is connected with decreased serotonin uptake of cells [47]. Moreover, the scales of depressive, anxious, cyclothymic and irritable temperaments were found to be closely associated in different populations suggesting real phenotypical connections beyond the similarities in the neurobiological background [48, 49]. Whether the clustering of dominant temperaments has any pronounced clinical relevance above single dominant temperaments is another question to be answered.

### Risk stratification of hypertensive patients

The calculation of total cardiovascular risk of hypertensive patients is a very important task of hypertension care [17]. Our results support the possibility that in the future the evaluation of affective temperaments might be the part of the risk stratification procedure of hypertensive patients as besides the obvious connection with mood disorders, the identification of DOM patients could determine a higher cardiovascular risk patient population as well.

### Limitations

The main limitation of our study is the relatively low number of patients involved. Out of 183 invited hypertensive patients, the arterial stiffness parameters of only 24 subjects with dominant affective temperament and 24 controls were analysed. The low number of patients limits the generalisability of our data and type I and type II errors cannot be excluded. As the detailed analysis of the impact of unique dominant affective temperaments on arterial stiffness, central blood pressure or seBDNF require higher number of involved patients, we defined this study as a “pilot”. Another limitation comes from the cross-sectional design of the study which precludes causal inference.

## Conclusions

In conclusion, although arterial stiffness parameters did not differ between the DOM subgroup of well-treated hypertensive patients and their hypertensive controls, their higher depressive and anxious scores and lower seBDNF might reflect their higher susceptibility for cardiovascular complications. Furthermore, DOM patients might bear a higher risk, as they have lower brachial and central diastolic blood pressure values in comparison to patients without dominant temperaments. The clinical significance of our findings with respect to the cardiovascular outcome of these patients needs further examinations on higher number of patients involved, but the cumulating data suggest that the evaluation of affective temperaments might improve both psychopathological and cardiovascular risk stratification.

## Abbreviations

AC: abdominal circumference
AI: augmentation index
ARBs: angiotensin II receptor blockers
BDI: beck depression inventory
BDNF: brain-derived neurotrophic factor
BMI: body mass index
Central DBP: central diastolic blood pressure
Central MBP: central mean blood pressure
Central PP: central pulse pressure
Central SBP: central systolic blood pressure
CKD-EPI GFR: glomerular filtration rate assessed by the chronic kidney disease epidemiology collaboration glomerular filtration rate equation
DBPB: diastolic brachial blood pressure
DOM: patients with dominant affective temperament
HAM-A: Hamilton Anxiety Scale
HR: heart rate
MBPB: mean brachial blood pressure
PPB: brachial pulse pressure
PWV: pulse wave velocity
SBPB: systolic brachial blood pressure
seBDNF: serum brain-derived neurotrophic factor
TEMPS-A: the temperament evaluation of Memphis Pisa, Paris and San Diego questionnaire
